# Systemic and local vascular inflammation and arterial reactive oxygen species generation in patients with advanced cardiovascular diseases

**DOI:** 10.3389/fcvm.2023.1230051

**Published:** 2023-09-07

**Authors:** Joanna Sulicka-Grodzicka, Piotr Szczepaniak, Ewelina Jozefczuk, Karol Urbanski, Mateusz Siedlinski, Łukasz Niewiara, Bartłomiej Guzik, Grzegorz Filip, Bogusław Kapelak, Karol Wierzbicki, Mariusz Korkosz, Tomasz J. Guzik, Tomasz P. Mikolajczyk

**Affiliations:** ^1^ Department of Rheumatology and Immunology, Jagiellonian University Medical College, Krakow, Poland; ^2^ School of Infection and Immunity, University of Glasgow, Glasgow, United Kingdom; ^3^ Department of Internal and Agricultural Medicine, Faculty of Medicine, Jagiellonian University Medical College, Krakow, Poland; ^4^ Center for Medical Genomics OMICRON, Jagiellonian University Medical College, Krakow, Poland; ^5^ Department of Interventional Cardiology, Jagiellonian University Medical College, John Paul II Hospital, Kraków, Poland; ^6^ Department of Cardiovascular Surgery and Transplantology, Jagiellonian University, John Paul II Hospital, Krakow, Poland; ^7^ BHF Centre for Research Excellence, Centre for Cardiovascular Sciences, The University of Edinburgh, Edinburgh, United Kingdom

**Keywords:** cardiovascular disease, cytokine, inflammation, oxidative stress, superoxide, hs-CRP

## Abstract

**Background:**

Systemic inflammation may cause endothelial activation, mediate local inflammation, and accelerate progression of atherosclerosis. We examined whether the levels of circulating inflammatory cytokines reflect local vascular inflammation and oxidative stress in two types of human arteries.

**Methods:**

Human internal mammary artery (IMA) was obtained in 69 patients undergoing coronary artery bypass graft (CABG) surgery and left anterior descending (LAD) artery was obtained in 17 patients undergoing heart transplantation (HTx). Plasma levels of tumor necrosis factor α (TNF-α), interleukin-6 (IL-6) and interleukin-1β (IL-1β) were measured using ELISA, high-sensitivity C-reactive protein (hs-CRP) was measured using Luminex, and mRNA expression of proinflammatory cytokines in the vascular tissues was assessed. Furthermore, formation of superoxide anion was measured in segments of IMA using 5 uM lucigenin-dependent chemiluminescence. Vascular reactivity was measured using tissue organ bath system.

**Results:**

TNF-α, IL-6 and IL-1β mRNAs were expressed in all studied IMA and LAD segments. Plasma levels of inflammatory cytokines did not correlate with vascular cytokine mRNA expression neither in IMA nor in LAD. Plasma TNF-α and IL-6 correlated with hs-CRP level in CABG group. Hs-CRP also correlated with TNF-α in HTx group. Neither vascular TNF-α, IL-6 and IL-1β mRNA expression, nor systemic levels of either TNF-α, IL-6 and IL-1β were correlated with superoxide generation in IMAs. Interestingly, circulating IL-1β negatively correlated with maximal relaxation of the internal mammary artery (*r* = −0.37, *p* = 0.004). At the same time the mRNA expression of studied inflammatory cytokines were positively associated with each other in both IMA and LAD. The positive correlations were observed between circulating levels of IL-6 and TNF-α in CABG cohort and IL-6 and IL-1β in HTx cohort.

**Conclusions:**

This study shows that peripheral inflammatory cytokine measurements may not reflect local vascular inflammation or oxidative stress in patients with advanced cardiovascular disease (CVD). Circulating pro-inflammatory cytokines generally correlated positively with each other, similarly their mRNA correlated in the arterial wall, however, these levels were not correlated between the studied compartments.

## Introduction

Atherosclerosis is known as a chronic inflammatory disease (1). Endothelial dysfunction, characterized by oxidative stress and increased expression of adhesion molecules and chemokines as well as reduced expression of nitric oxide, is regarded as a pivotal stage in the initiation of atherogenesis (2). Oxidation inherently interacts with inflammation in the pathogenesis of endothelial dysfunction. Leukocyte adherence and infiltration into the vessel wall (3) has been shown to stimulate vascular reactive oxygen species (ROS) production by both infiltrating immune cells and activated vascular cells (4, 5). Increased superoxide production is a critical step in endothelial dysfunction (4, 6–9). At the same time low grade inflammation is a hallmark of cardiovascular disease (CVD). Recent clinical trials including CANTOS [Canakinumab Anti-inflammatory Thrombosis Outcome Trial (10)], as well as COLCOT (Colchicine Cardiovascular Outcomes Trial) and LoDoCo2 (low-dose colchicine trial) (11, 12) have demonstrated proof of principle of potential targeting of the inflammatory mechanisms in cardiovascular risk. These are supported by mechanistic evidence in murine and human studies linking systemic inflammation to local events in the vessel wall (13).

Inflammation can be a cause and a consequence of heart failure (HF), and it contributes to disease pathogenesis and progression. The hemodynamic stress associated with HF induces inflammation by eliciting the release of proinflammatory cytokines by cardiomyocytes and cardiac fibroblasts, including TNF-α, IL-6, IL-1β as well as angiotensin II, and myostatin. HF also causes mitochondrial dysfunction, reactive oxygen species generation, and leads to activation of the NLRP3 inflammasome, with subsequent production of proinflammatory cytokines (14–16). The proinflammatory cytokines IL-1β and TNF-α may induce systolic and diastolic dysfunction, TNF-α also stimulates cardiac remodeling (17). Additionally, comorbidities that commonly coexist with HF including diabetes, obesity, and chronic kidney disease are associated with chronic low-grade inflammation, which may have detrimental effects on cardiac structure and function (18). A recently published analysis of the CANTOS trial found that, anti-IL-1β therapy was associated with a significant reduction in HF hospitalizations and a reduction in the composite of HF hospitalizations and all-cause death, in participants who had a reduction in hs-CRP to <2 mg/L compared with placebo (19).

In light of these relationships, it becomes essential to identify biomarkers that could reflect vascular inflammation as well as related pathogenetic mechanisms of vascular dysfunction (20–22). While increased levels of circulating biomarkers such as IL-6 as well as hs-CRP independently predict the risk of cardiovascular diseases (23, 24), it remains unclear to what extent they represent local processes occurring in the vessel wall.

Accordingly, as there are regional differences in arterial vessels in susceptibility to atherosclerosis, and different pathological pathways might be involved in different vessels, yet some systemic markers of inflammation have been reported to be associated with increased risk of CVD. We sought to investigate whether levels of circulating inflammatory cytokines (TNF-α, IL-6 and IL-1β) reflect the local vascular expression of these inflammatory molecules in different types of blood vessels (internal mammary artery and left anterior descending artery) of patients with advanced cardiovascular diseases [coronary artery disease patients undergoing coronary artery bypass graft (CABG) surgery, and patients with end-stage heart failure undergoing heart transplantation]. We also investigated whether oxidative stress is associated with inflammation in atherosclerosis resistant artery (internal mammary artery) in patients with coronary artery disease (CAD). We chose these blood vessels as a model as they represent different aspects of the spectrum of susceptibility to atherosclerosis. While human internal mammary arteries do not develop atherosclerosis in spite of overt endothelial dysfunction, coronary arteries are an essential site of atherosclerosis development (25).

## Materials and methods

### Study participants

The study population consisted of 69 patients undergoing coronary artery bypass graft (CABG) surgery and 17 patients undergoing heart transplantation (HTx). Exclusion criteria included active infections, inflammatory, neoplastic, or hepatic disorders (26). Anthropometric measurements were recorded, and body mass index (BMI) was calculated. Blood pressure measurements were performed using validated devices. Each measurement was performed twice with the participant in a seated position, after at least 5 min of rest. Hypertension was defined as blood pressure ≥140/90 mmHg or taking antihypertensive medications. Smoking status was defined as past-smokers, those who had stopped cigarette smoking for ≥6 months prior to surgery, or current smokers. Dilated cardiomyopathy (DCM) diagnosis was based on morphology criteria when elevated left ventricular end-diastolic diameter (LVEDd) and systolic dysfunction were reported in transthoracic echocardiography. Ischemic heart disease (IHD) diagnosis was established either based on the presence of significant coronary lesions in recent coronary angiography, any history of prior coronary revascularization, or a history of myocardial infarction. Hypertrophic cardiomyopathy (HCM) and arrhythmogenic right ventricular cardiomyopathy (ARVCM) diagnoses were reported only when they were explicitly present in prior medical history. Otherwise, when a systolic dysfunction was present in echocardiography, but no specific criteria for the aforementioned cardiomyopathies were met, the patients were identified as idiopathic cardiomyopathy. Written informed consent was obtained from all patients. The study was approved by local Bioethics Committee of the Jagiellonian University (KBET124/B/2012 and Approval No. 122.6120.46.2017 and 1072.6120.162.2019).

### Human arterial sampling

Tissue samples of internal mammary artery (IMA) were obtained during CABG surgery using the no-touch technique prior to surgical distension, as previously described (26). Tissue samples of the left anterior descending coronary artery (LAD) were obtained during heart transplantation. All samples were received from the Department of Cardiovascular Surgery and Transplantology of the John Paul II Hospital in Krakow (Poland). IMA and LAD samples were immediately transferred to ice-cold Hank's Balanced Salt Solution (Gibco; Thermo Fisher Scientific) buffer and were maintained at 4°C (27). The surrounding tissues were separated from the vessels using microsurgical instruments and a microscope.

### Gene expression measurements in human arteries

The isolated blood vessels were stored in a RNA-later stabilisation solution (Ambion, Thermo Fisher Scientific) until RNA isolation. The tissue was homogenised for 20 min in TRI reagent solution (Thermo Fischer Scientific) using the TissueLyser LT bead mill (Qiagen, USA). Total RNA was isolated from the samples using the Direct-zol RNA Miniprep kit (Zymo Research) according to the manufacturer's protocol. Reverse transcription was performed using 500 ng of RNA using the High Capacity cDNA reverse transcription kit (Applied Biosystems, USA). Real-time PCR reactions were performed on the 7900HT instrument (Applied Biosystems, USA) using commercially available TaqMan assays for IL-1β (Hs01555410_m1), IL-6 (Hs00174131_m1), TNF (Hs00174128_m1). Data were normalized to levels of Eukaryotic Translation Elongation Factor 2 (EEF2) (Hs00157330_m1) mRNA, and then dCT was calculated.

### Lucigenin-enhanced chemiluminescence

The level of vascular superoxide production was evaluated by lucigenin-dependent chemiluminescence (LGCL) as previously described (26, 28). The vessels were cut to expose the endothelium and then aerated with 95% O2% and 5% CO2 in Krebs-HEPES buffer for 20 min. Measurements were made with 5 µM lucigenin (Millipore Sigma) in Krebs-HEPES buffer using a FB12 chemiluminometer (Berthold). ROS production was given in RLU per second per milligram of dry weight of the vessel (28).

### Vascular reactivity measurements

Isometric tension studies were performed using tissue organ bath system 750TOBS (Danish Myo Technology) as previously described (4, 26). Segments of IMA rings were equilibrated in Krebs-Henseleit solution (in mM: 124 NaCl, 4.6 KCl, 2.5 CaCl2, 1.2 MgSO4, 1.2 KH2PO4, 0.01 EDTA, 23 NaHCO3, and 11 glucose), with continuous gas (CO_2_ 5%/O_2_ 95%) and checked for viability with KCl (60 mN). Following pre-constriction with phenylephrine (Phe) (up to 70% of the maximum KCl contraction), the vessels were relaxed with acetylcholine (ACh) and sodium nitroprusside (SNP) to obtain endothelium-dependent (ACh) and endothelium-independent (SNP) vasodilatations.

### Biomarker assessment

Peripheral blood was collected in tubes containing ethylenediaminetetraacetic acid (EDTA) prior to the premedication for the surgery. Within 1 h after blood collection, samples were centrifuged at 400×g for 10 min. The platelet-rich plasma was then collected and centrifuged at 2,000×g for 15 min at 4°C. Plasma samples without pellets were then collected and stored at −80°C until analysis. The concentration of selected cytokines in plasma was measured using commercially available high-sensitivity (HS) ELISA kits for Human IL-1β/IL-1F2 (HSLB00D), IL-6 (HS600C) and TNF-α (HSTA00E). All kits were purchased from R&D Systems (Bio-Techne) and performed according to the manufacturer's instructions. The final absorbance readings were obtained using a Synergy H4 Hybrid Multi-Mode Microplate Reader (Thermo Fisher Scientific, Waltham, MA, USA). Plasma samples were also analysed for hs-CRP (sensitivity 0.024 µg/ml) with Luminex technology using a commercial assay (Human Luminex® Discovery Assay**,** LXSAHM**,** R&D) and were read on a Luminex 200 System (Merck, Millipore) in accordance with the manufacturer's instructions.

### Statistical analysis

The deviation from normal distribution of variables was assessed using Shapiro-Wilk test. The continuous variables are presented as mean ± SD, whereas the categorical variables are presented as numbers and percentages. Continuous variables with non - normal distribution are presented as median (25th; 75th percentile). Continuous variables were compared using the Student's *t*-test. Pearson product-moment correlations were performed to analyse the correlation between IL-1β, IL-6 or TNF-α mRNA expression in the arteries and their corresponding plasma levels and hs-CRP. Sensitivity analyses were performed using non-parametric Spearman's rank order correlations and Mann–Whitney *U-*tests. This has been performed to assure results were not driven be skewed distribution of the variables. Spearman's rank order correlations were performed to analyse the correlation between vascular superoxide production, maximal relaxation to acetylcholine, and circulating or vascular proinflammatory cytokines. Multivariable regression analysis adjusting for age, BMI, sex and current smoking was used to determine the association between circulating inflammatory cytokines. These analyses used log10-transformed cytokine levels and normal distribution of regression-derived residuals was confirmed using Shapiro–Wilk test. A *p*-value below 0.05 was considered as statistically significant. All analyses were performed using IBM SPSS Statistics (New York, United States) package (version 28.0.1.0) and graphs were drawn using Graph-Pad Prism v9.5.1. A correlation matrix was performed using Statistical tools for high-throughput data analysis (STHDA, http://www.sthda.com/english/rsthda/correlation-matrix.php).

## Results

### Patients' characteristics

The clinical characteristics of the study participants in whom IMAs and human coronary arteries (LAD) were obtained are presented in Tables 1, 2, respectively. The mean age of patients undergoing CABG was 63.6 ± 8.8 years, and 68% of patients were males. Among patients undergoing cardiac transplantation, who were aged 50.2 ± 10.6 years, 94% were males. Ischemic heart disease (IHD) and dilatated cardiomyopathy (DCM) were diagnosed in 58.8% and 64.7% of patients with end-stage heart failure undergoing HTx. IL-1β plasma level was positively associated with hypertension, whereas the circulating level of IL-6 was positively correlated with the age in CABG cohort (Table 3 and Supplementary Table S1).

**Table 1:** Clinical characteristics of patients undergoing CABG surgery.

Variables	*n* = 69
Age, years	63.6 ± 8.8
Male, *n* (%)	47 (68.1)
SBP (mmHg)	137.4 ± 18
DBP (mmHg)	79.9 ± 8.8
Risk factors	
Hypertension, *n* (%)	65 (94.2)
Hypercholesterolemia, *n* (%)	57 (82.6)
BMI, kg/m^2^	28.5 ± 3.9
Smokers, *n* (%)	
Overall	40 (58.0)
Current	19 (27.5)
Diabetes mellitus, *n* (%)	28 (40.6)
Coronary angiography	
Three-vessel disease, *n* (%)	39 (56.5)
Two-vessel disease, *n* (%)	27 (39.1)
One vessel disease, *n* (%)	0 (0)
Medications	
Aspirin, *n* (%)	60 (87.0)
P2Y12 inhibitors, *n* (%)	21 (30.4)
Statins, *n* (%)	60 (87.0)
ACEI, *n* (%)	52 (75.4)
ARB, *n* (%)	7 (10.1)
Diuretics, *n* (%)	11 (15.9)
β-blockers, *n* (%)	55 (79.7)
Ca-blockers, *n* (%)	16 (23.2)
Insulin, *n* (%)	7 (10.1)
Oral antidiabetics, *n* (%)	15 (21.7)
Biomarker concentration	
TNF-α (pg/ml)	1.33 (1.12; 1.65)
IL-6 (pg/ml)	3.10 (1.64; 5.01)
IL-1β (pg/ml)	0.37 (0.18; 0.55)
hs-CRP (μg/ml)	1.39 (0.56; 3.56)

Values are presented as mean ± standard deviation (SD) or median (25th; 75th percentile) or *n* (%).

ACEIs, angiotensin-converting enzyme inhibitors; ARBs, angiotensin II receptor blockers; BMI, body mass index; Ca, calcium channel; CABG, coronary artery bypass graft; DBP, diastolic blood pressure; hs-CRP, high sensitivity C reactive protein; IL,- interleukin; SBP, systolic blood pressure; TNF, tumor necrosis factor.

**Table 2:** Clinical characteristics of patients undergoing HTx.

Variables	*n* = 17
Age, years	50.2 ± 10.6
Male, *n* (%)	16 (94.1)
DCM, *n* (%)	11 (64.7)
HCM, *n* (%)	3 (17.6)
ARVCM, *n* (%)	1 (5.9)
Idiopathic CM	2 (11.8)
IHD, *n* (%)	10 (58.8)
SBP, mmHg	104.3 ± 11.2
DBP, mmHg	71.8 ± 8.6
Risk factors	
Hypercholesterolemia, *n* (%)	8 (47.1)
BMI, kg/m^2^	24.3 ± 4.2
Current smokers, *n* (%)	2 (11.8)
Diabetes mellitus, *n* (%)	2 (11.8)
Kidney disease, *n* (%)	2 (11.8)
Medications	
Aspirin, *n* (%)	2 (11.8)
Statins, *n* (%)	5 (29.4)
ACEI, *n* (%)	4 (23.5)
ARB, *n* (%)	3 (17.6)
Diuretics, *n* (%)	7 (41.2)
β-blockers, *n* (%)	6 (35.3)
Ca-blockers, *n* (%)	0 (0)
Biomarker concentration	
TNF-α (pg/ml)	1.38 (0.99; 2.19)
IL-6 (pg/ml)	5.02 (2.89; 8.73)
IL-1β (pg/ml)	0.07 (0.05; 0.11)
hs-CRP (μg/ml)	0.66 (0.09; 4.49)

Values are presented as mean ± standard deviation (SD) or median (25th; 75th percentile) or *n* (%).

ACEIs, angiotensin-converting enzyme inhibitors; ARBs, angiotensin II receptor blockers; ARVCM, arrhythmogenic right ventricular cardiomyopathy; BMI, body mass index; Ca, calcium channel; CM, cardiomyopathy; DBP, diastolic blood pressure; DCM, dilated cardiomyopathy; HCM, hypertrophic cardiomyopathy; hs-CRP, high sensitivity C reactive protein; HTx, heart transplantation; IHD, ischemic heart disease; IL, interleukin; SBP, systolic blood pressure; TNF, tumor necrosis factor.

**Table 3:** Significance level of the differences in association between vascular and plasma markers with risk factors in CABG cohort.

	Risk factors					
	Age	Sex	Current smoking	Diabetes	Hypertension	Obesity
Vascular mRNA expression (dCT)						
TNF	0.394	0.678	0.710	0.408	0.531	0.884
IL-6	0.253	0.949	0.684	0.546	0.150	0.090
IL-1β	0.969	0.836	0.740	0.762	0.160	0.685
Plasma protein level						
TNF	0.386	0.342	0.231	0.304	0.071	0.685
IL-6	0.045*	0.042*	0.244	0.298	0.754	0.297
IL-1β	0.071	0.395	0.095	0.642	0.017*	0.576

*p*-value of the association between vascular and circulating markers with risk factors including age, sex, current smoking, diabetes, hypertension, and obesity is shown. The analysis was performed using Pearson product-moment correlation for the association between age and both vascular and plasma markers. *T*-test was used for other risk factors including sex, current smoking, diabetes, hypertension, and obesity. Pearson product-moment correlation revealed the positive correlation between plasma level of IL-6 and age (*r* = 0.242, *p* < 0.05). Circulating IL-6 level was higher in men than in women (4.25 ± 3.53 vs. 2.96 ± 1.64, *p* < 0.05).

* *p*<0.05.

### Plasma biomarker concentrations and their vascular mRNA expression

**Figure 1:**
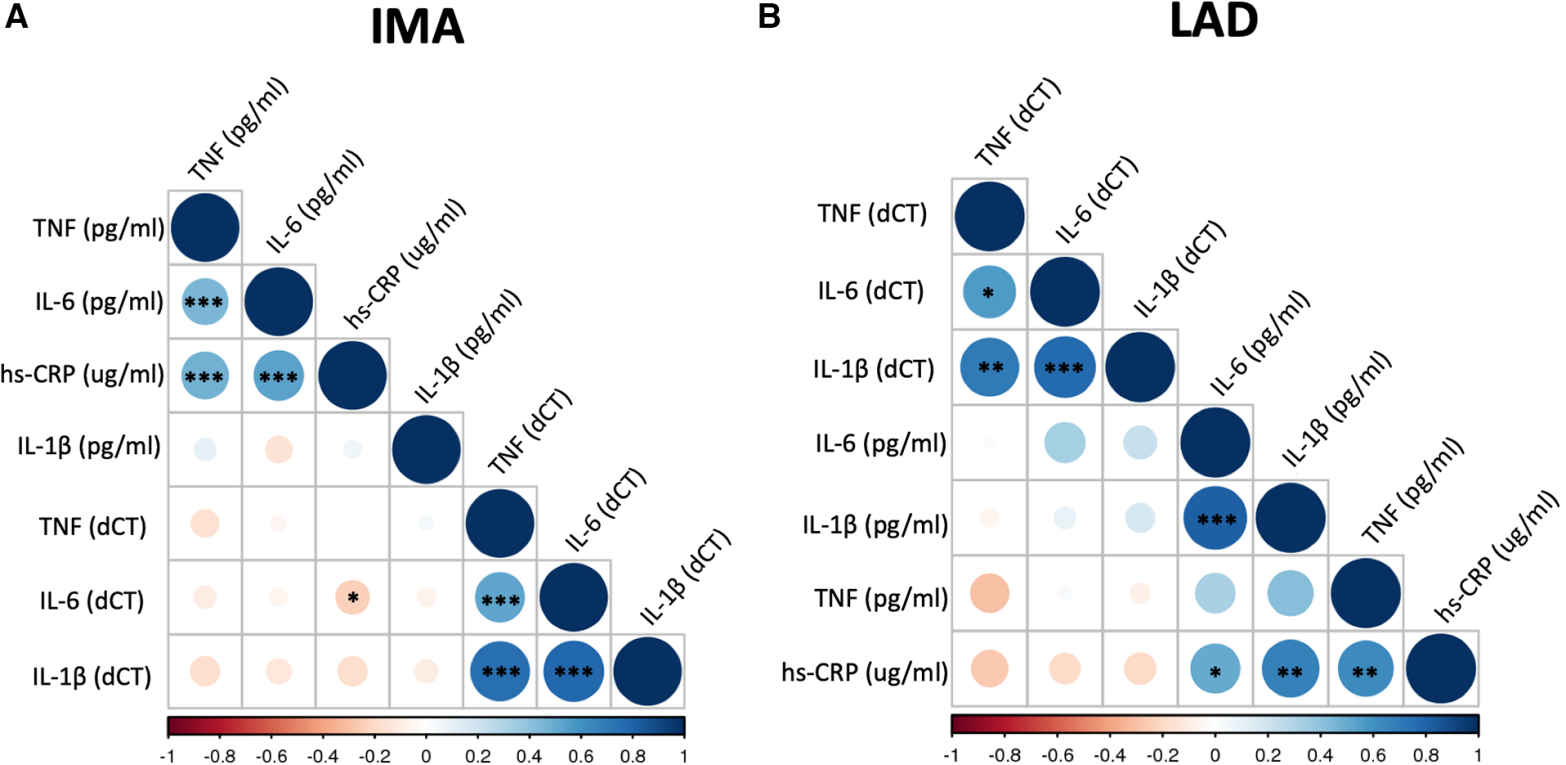
Correlation matrix of mRNA expression of selected biomarkers in blood vessels and their plasma levels. Correlation matrix shows the relationship between mRNA expression of TNF (TNF-α), IL-6, and IL-1β in the internal mammary arteries (IMA) (**A**) and left anterior descending coronary arteries (LAD) (**B**) and their plasma concentrations as well as hs-CRP level. Samples were obtained from patients undergoing CABG surgery (**A**) or heart transplantation (HTx) (**B**). The correlations were calculated using Pearson product-moment correlation. Colour and its intensity indicate r values i.e., direction and strength of correlation (blue positive correlation, red negative correlation), as per X axis. *p < 0.05, **p < 0.01, ***p < 0.001.

The mRNA expression of TNF-α, IL-1β and IL-6 in the blood vessels was not correlated with plasma protein levels in both CABG and HTx groups (Figures 1A,B, Supplementary 1SA,1SB,2SA,2SB and Supplementary Table S2). The mRNA expression of all proinflammatory cytokines positively correlated with each other, both within IMA and LAD (Figures 1A,B and Supplementary Figure 2S). Additionally, we observed a positive correlation between the level of IL-6 and TNF-α (*r* = 0.45, *p* < 0.001 for Pearson product-moment correlation) in plasma collected from CABG patients (Figure 1A and Supplementary Figure 2SA), which remained significant in a multivariable linear regression analysis after adjustment for sex, age, BMI and current smoking status (*B* = 0.184 log10 (TNF-α)/llog10 (IL-6), SE = 0.051, *p* < 0.001). In the HTx patients we found a strong positive correlation between the plasma level of IL-6 and IL-1β (*r* = 0.82, *p* < 0.0001) (Figure 1B), which remained significant after adjustment for age and BMI in a multivariable analysis (*B* = 0.85 log10 (IL-1β)/llog10 (IL-6), SE = 0.29, *p* = 0.02). The positive corelations between hs-CRP and TNF-α and IL-6 have been observed in CABG cohort (Figure 1A and Supplementary Figure 2SA) and remained significant after adjustment for sex, age, BMI and current smoking status (respectively *B* = 1.60 log10 (hs-CRP)/llog10 (TNF-α), SE = 0.44, *p* < 0.001 and *B* = 1.08 log10 (hs-CRP)/llog10(IL-6), SE = 0.16, *p* < 0.001).There was a modest negative correlation between plasma hs-CRP and mRNA expression (dCT) of IL-6 in IMA (*p* < 0.05) (Figure 1A). However, this was not significant when Spearman's rank order correlation was used (Supplementary Figure 2S). In HTx group, hs-CRP positively correlated with TNF-α (Figure 1B and Supplementary Figure 2SB).

### Inflammatory cytokines and vascular oxidative stress

**Figure 2:**
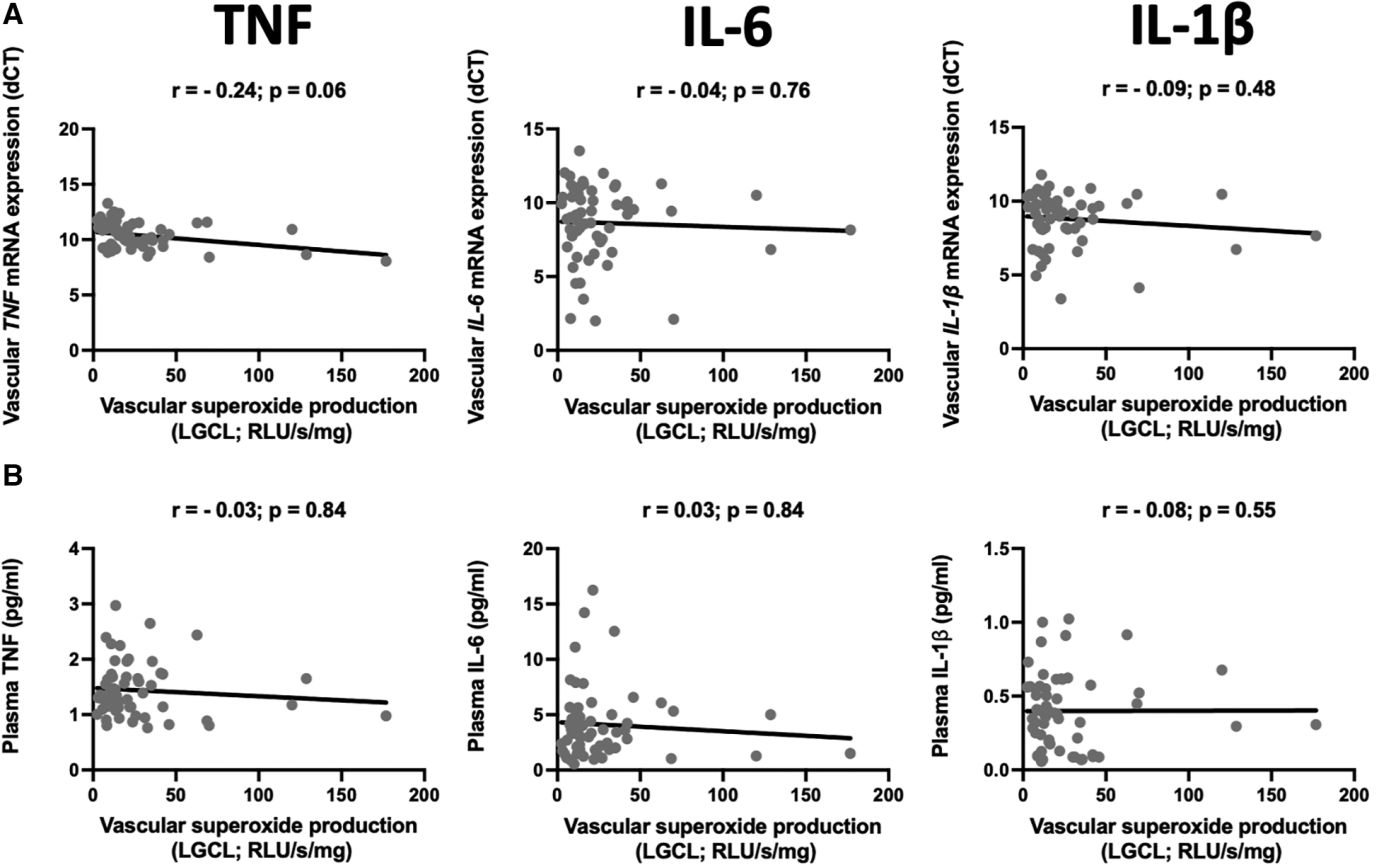
Relationship between mRNA expression and plasma levels of selected pro-inflammatory cytokines and superoxide production. The scatter plots show the correlations between mRNA expression of TNF (TNF-α), IL-6, and IL-1β in the internal mammary arteries (IMA) (**A**) and their plasma levels (**B**) and vascular superoxide production measured by the lucigenin-dependent chemiluminescence (LGCL). Samples were obtained from patients undergoing CABG surgery. The correlations were calculated using Spearman's rank order correlation. *N* = 59.

We did not observe any statistically significant correlations between superoxide production and vascular expression of TNF-α, IL-6 or IL-1β (Figure 2A). Circulating levels of these cytokines were not correlated to superoxide production in *ex vivo* vascular segments, measured by low concentration of lucigenin enhanced chemiluminescence (Figure 2B).

### Inflammatory cytokines and blood pressure

In the next step, we performed the additional analysis of the mRNA expression of TNF-α, IL-6 and IL-1β in the blood vessels and their plasma protein levels and both systolic or diastolic blood pressure (SBP, DBP, respectively) in CABG group. We observed that none of the studied circulating cytokines correlated with SBP or DBP (Table 4 and Supplementary Table S3). However, we observed the positive correlation between vascular expression of TNF-α (dCT) or IL-6 (dCT) and DBP (Table 4 and Supplementary Table S3). We did not find any significant correlation between vascular superoxide production and systolic or diastolic blood pressure (Table 4).

**Table 4:** Relationship between mRNA expression and plasma levels of selected pro-inflammatory cytokines, vascular superoxide, and blood pressure values.

	SBP	DBP
Vascular mRNA expression (dCT)		
TNF	0.07	0.27*
IL-6	0.11	0.27*
IL-1β	0.12	0.23
Plasma protein level		
TNF	0.11	0.19
IL-6	0.01	0.09
IL-1β	0.06	0.15
Vascular superoxide production	-0.03	−0.03

Correlations (*r*) between mRNA expression of TNF (TNF-α), IL-6, IL-1β in the internal mammary arteries (IMA) and their plasma levels, vascular superoxide and systolic or diastolic blood pressure (SBP, DBP, respectively). Samples were obtained from patients undergoing CABG surgery. The correlations were calculated using Pearson product-moment correlation (for dCT and plasma protein level) or Spearman's rank order correlation (for vascular superoxide production).

* *p* < 0.05.

### Endothelial dysfunction in CABG is associated with plasma Il-1β level

**Figure 3:**
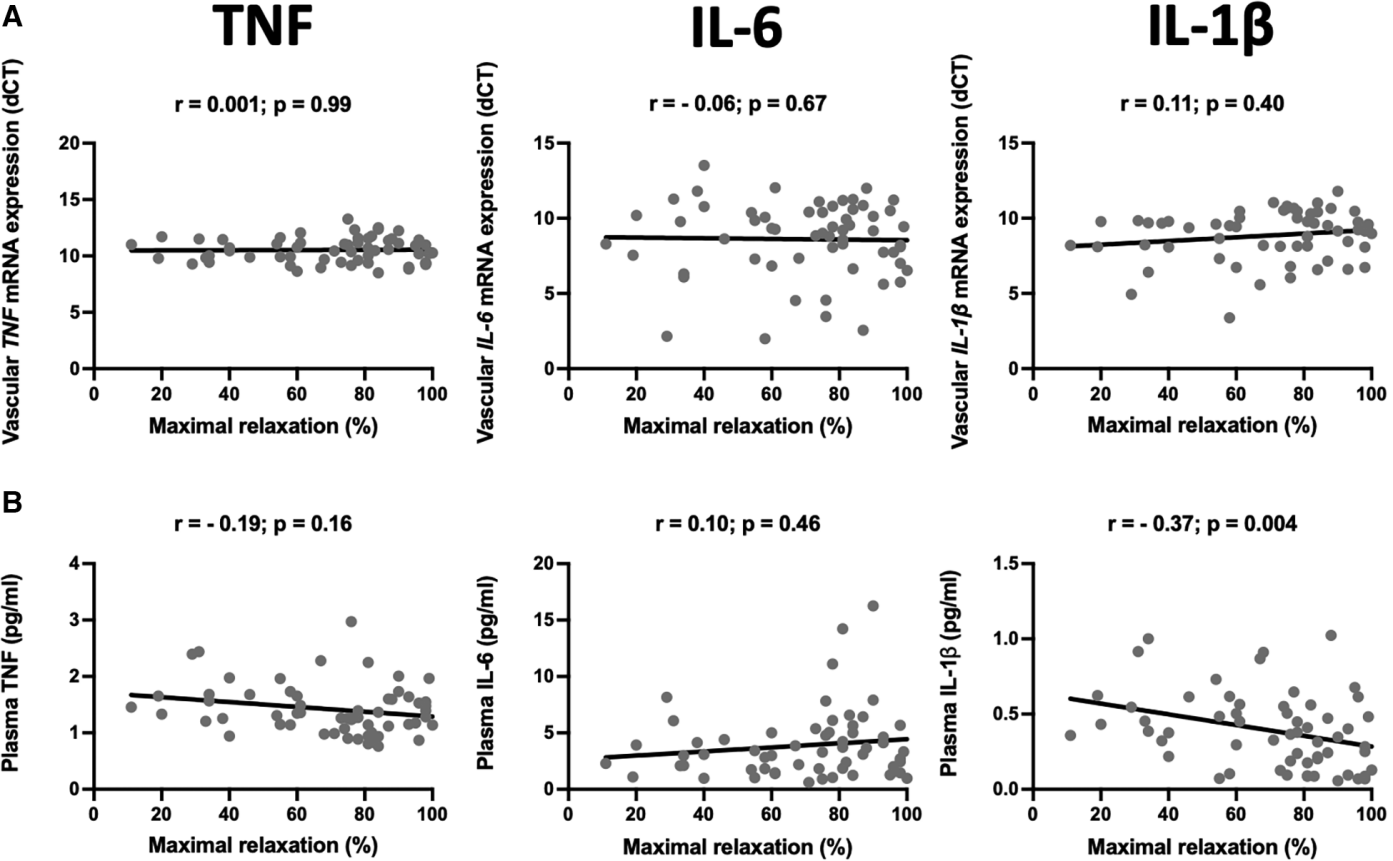
Relationship between mRNA expression and plasma levels of selected pro-inflammatory cytokines and endothelial function. The scatter plots show the correlations between mRNA expression of TNF (TNF-α), IL-6, and IL-1β in the internal mammary arteries (IMA) (**A**) and their plasma levels (**B**) and endothelial function measured as a maximal relaxation to acetylcholine. Samples were obtained from patients undergoing CABG surgery. The correlations were calculated using Spearman's rank order correlation. *N* = 59.

Atherosclerosis has the heterogenous nature. Although the internal mammary arteries are resistant to the development of atherosclerosis, we observed that some of the vessels are characterized by endothelial dysfunction measured as a relaxation to acetylcholine. Interestingly, from all studied vascular and plasma cytokines, circulating IL-1β negatively correlated with the maximal relaxation (*r* = −0.37, *p* = 0.004, Figure 3).

## Discussion

In this study we demonstrate that the levels of systemic TNF-α, IL-1β and IL-6 do not reflect their vascular mRNA expression in patients with advanced cardiovascular diseases, and that vascular expression of these cytokines is not associated with increased ROS generation in IMA in patients with coronary artery disease. Interestingly, we found that proinflammatory cytokine TNF-α positively correlated with high sensitivity C-reactive protein in patients with advanced heart failure undergoing heart transplantation. In CABG cohort the positive correlation of hs-CRP with TNF-α and IL-6 was observed. Serum CRP is considered as a marker of inflammation as well as a predictor of the development of cardiovascular events (29, 30). Our results are consistent with previous published data. It has been shown that human coronary artery smooth muscle cells are able to produce CRP in response to proinflammatory cytokines including TNF-α, IL-1β and IL-6 (29). Interestingly, locally produced CRP participated in the atherogenesis (29, 30). CRP was increased in patients with coronary artery lesions. Moreover, CRP positively correlated with IL-6 (31–33) and TNF-α (33–35).

In our study we observed no correlation between systolic blood pressure and systemic or vascular inflammatory cytokines. However, we found the correlation between diastolic blood pressure and vascular expression of TNF-α and IL-6. Previously, it has been shown that IL-6 and TNF-α, positively correlated with blood pressure in pregnancy induced hypertension (36, 37). Moreover, the positive correlation between IL-1β and mean blood pressure in patients with essential hypertension has been demonstrated (38). However, it should be noted that IL-1β inhibition by canakinumab reduces adverse cardiovascular events but this mechanism is not associated with changes in blood pressure (39). These discrepancies may be related to the fact that patients in our study had more advanced CVD, and comorbidities and medications may influence the relationship between blood pressure and inflammatory markers.

Previous studies have confirmed the role of TNF-α in vascular pathology (40, 41). TNF-α is synthesized initially by activated macrophages and T cells, it acts by binding to one of two types of receptors: TNFR1 or TNFR2 (tumor necrosis factor receptor) (42), and employs the stimulation of the release of IL-1β and IL-6, as well as upregulation of the expression of chemokines and endothelial adhesion molecules, and coordination of the migration of leukocytes to the inflamed targeted organs (40). In humans, evidence points towards TNF-α playing an important role in stimulating endothelial dysfunction. The positive correlation between increased plasma TNF-α levels and reduced reactivity to acetylcholine has been observed previously (41). In diabetic patients, TNF-α induces endothelial dysfunction, which is reversed by the PPAR-γ agonist (43). Moreover, the TNF-α inhibitors are particularly effective in blood pressure lowering in animal studies (44). ROS appears to be one of the key mediators involved in TNF-α induced endothelial activation. Mycophenolic acid, active metabolite of mycophenolate mofetil inhibits TNF-α-stimulated ROS generation in human aortic endothelial cells (45). TNFRs activate transcription factor NF-κB (nuclear factor-κB). ROS also function as second messengers in several signal transduction pathways including NF-κB signaling (46). ROS trigger activation of NF-κB but may also inhibit NF-κB activity. These opposite effects seem to be time and context dependent (47). In our study the vascular TNF expression (dCT) in IMA negatively correlated with vascular superoxide production, but this interaction did not reach the statistical significance. No correlation of TNF with maximal relaxation was found. Interestingly, we observed the strong negative correlation of plasma IL-1β with the maximal relaxation. This observation is consistent with previous study showing the link between IL-1β and coronary endothelial dysfunction (48).

Discordance between levels of systemic cytokines and their vascular expression may be partially explained by the complexity of the inflammatory process. Cytokines are local mediators, and their production can be compartmentalized (49). Patterns of cytokine production differ under various clinical conditions, particularly in low-grade chronic inflammation, and cellular mRNA may not correspond to circulation proteins. Although elevations in inflammatory markers commonly occur together, they may not be synchronized. Additionally, concentrations of some inflammatory markers change relatively slowly, while other can change rapidly. Accurate detection of cytokines is challenging because of their dynamic secretion processes and short half-lives. The majority of cytokines have a short half-life *in vivo* and may be subjected to immediate degradation during sample collection and preparation (50).

Interestingly, multiple distinct endothelial cells subtypes have been revealed in aorta by single-cell sequencing (51). At the same time, vascular dysfunction can correlate between different arteries, as well as between venous and arterial endothelium, suggesting that endothelial dysfunction may be systemic (52). Furthermore, genetic and epigenetic modifications may influence endothelial function both in healthy individuals and in patients with cardiovascular diseases (53). Immune response can also vary within the individual, between local and systemic scales and across the tissues.

The analysis of biomarkers in patients with atrial fibrillation (AF) has revealed compelling associations between various mechanisms and cardiovascular death. These mechanisms include oxidative stress, cardiorenal dysfunction, inflammation, vascular and renal dysfunction, calcium balance, apoptosis, and fibrinolysis (54). Additionally, the associations between biological processes related to IFN-γ production, T-cell co-stimulation, and all-cause mortality have been demonstrated. In light of these findings, the potential therapeutic targets may involve enhancing IFN-γ production and targeting CD28, CD70, TNF superfamily member-14 (TNFSF14), and inducible costimulator ligand (ICOSLG) (55). Furthermore, it is worth noting that circulating cardiovascular disease biomarkers may also be associated with an increased risk of cancer and overall mortality (56).

Our study does have certain limitations. Firstly, we have evaluated the association of plasma biomarker concentrations and their vascular mRNA expression but not vascular protein level. It should be noted that during the surgery there were collected very small fragments of the vessel, and we were unable to perform the additional analyses, including the measurements of cytokine vascular protein levels. This may be the limitation of our study. On the other hand, cytokines are small, secreted proteins, which are produced by immune cells as well as a number of vascular cells (57). For this reason, the vascular cytokine mRNA expression may be more representative than its vascular protein level. Moreover, examining mRNA levels could offer valuable insights into the interplay between inflammation and oxidation, as ROS may regulate cytokine transcription in vascular cells. In addition, no histological data were available on the human vessels.

Secondly, it is important to note that the patients included in our study had advanced CVD and multimorbidity, which indicates the presence of several potential confounding factors that could influence the systemic levels of peripheral inflammatory markers more than local vascular expression. These confounders may include factors such as adipose tissue in obesity, medication usage, and other comorbidities. We do not have data on the imaging of other arteries which could show the extent of atherosclerosis. Although, it may be speculated that presence of clinical atherosclerosis in one vascular territory largely indicates an increased likelihood of its presence in other vessels.

Another limitation is a relatively small number of HTx patients included for this study. We have collected 17 samples of the left anterior descending artery from HTx cohort. However, it should be noted that, other studies on the human cardiac tissue and coronary arteries were performed on a smaller number of samples, collected from 10 to 14 patients (27, 58, 59).

Furthermore, our study involved a single systemic cytokine measurement. This may partially explain the lack of relationship observed with local production, as systemic levels can vary depending on environmental and other factors. Therefore, our analysis may not fully capture the potential variability over time. However, it is worth noting that in practical terms, our aim was to identify biomarkers that could be implemented clinically without the need for multiple consecutive measurements. Requiring frequent measurements could hinder their feasibility and practical use in a clinical setting.

In conclusion, our findings suggest that while individual cytokines show correlation within their respective compartments, indicating consistency in local processes, we observed a lack of correlation between cytokine levels across compartments. This lack of correlation between local cytokine expression and their circulating levels highlights the complex nature of inflammation, which may involve intricate aspects that cannot be fully captured by measuring circulating inflammatory cytokines.

## Data Availability

The original contributions presented in the study are included in the article/**Supplementary Material**, further inquiries can be directed to the corresponding author.
