# Supplementary material for: Systemic and local vascular inflammation and arterial reactive oxygen species generation in patients with advanced cardiovascular diseases
Source: Front Cardiovasc Med. 2023 Sep 7;10:1230051. doi: 10.3389/fcvm.2023.1230051 (PMC10513373; doi:10.3389/fcvm.2023.1230051)
Supplement: Supplementary file 1 [file Datasheet1.pdf]

Systemic and local vascular inflammation and arterial reactive oxygen species generation in patients with advanced cardiovascular diseases

Joanna Sulicka-Grodzicka[1,2], Piotr Szczepaniak[3,4], Ewelina Jozefczuk[3,4], Karol Urbanski[3], Mateusz Siedlinski[3,4], Łukasz Niewiara[5], Bartłomiej Guzik[5], Grzegorz Filip[6], Bogusław Kapelak[6], Karol Wierzbicki[6], Mariusz Korkosz[1], Tomasz J. Guzik[3,4,7], Tomasz P. Mikolajczyk[2,3,4]

[1] Department of Rheumatology and Immunology, Jagiellonian University Medical College, Krakow, Poland

[2] School of Infection and Immunity, University of Glasgow, Glasgow, UK

[3] Department of Internal and Agricultural Medicine, Faculty of Medicine, Jagiellonian University Medical College, Krakow, Poland

[4] Center for Medical Genomics OMICRON, Jagiellonian University Medical College, Krakow, Poland

[5] Department of Interventional Cardiology, Jagiellonian University Medical College, John Paul II Hospital, Kraków, Poland

[6] Department of Cardiovascular Surgery and Transplantology, Jagiellonian University, John Paul II Hospital, Krakow, Poland

[7] BHF Centre for Research Excellence, Centre for Cardiovascular Sciences, The University of Edinburgh, Edinburgh, UK

Running title: Inflammation in cardiovascular diseases

*** Correspondence:**

Tomasz P. Mikolajczyk, PhD

email: tomaszp.mikolajczyk@uj.edu.pl
Translational Medicine Laboratory
Department of Internal and Agricultural Medicine,
Jagiellonian University Medical College,
Skarbowa 1
31-121 Krakow, Poland
Tel. +48 12 633 00 03
Fax. +48 12 687 62 12

Keywords: Cardiovascular disease, Cytokine, Inflammation, Oxidative stress, Superoxide

Table 1 Supplement. Significance level of the differences in association between vascular and plasma markers with risk factors in CABG cohort.

	Risk factors					
	Age	Sex	Current smoking	Diabetes	Hypertension	Obesity
**Vascular mRNA expression (dCT)**						
**TNF**	0.385	0.440	0.638	0.386	0.538	0.874
**IL-6**	0.462	0.410	0.851	0.990	0.095	0.094
**IL-1β**	0.822	0.537	0.528	0.392	0.111	0.765
**Plasma protein level**						
**TNF**	0.546	0.098	0.330	0.587	0.032*	0.188
**IL-6**	0.035*	0.226	0.147	0.816	0.837	0.642
**IL-1β**	0.089	0.300	0.156	0.976	0.009**	0.472

P value of the association between vascular and circulating markers with risk factors including age, sex, current smoking, diabetes, hypertension, and obesity is shown. The analysis was performed using Spearman's rank order correlation for the association between age and both vascular and plasma markers. Mann-Whitney U test was used for other risk factors including sex, current smoking, diabetes, hypertension, and obesity. Spearman's rank order correlation revealed the positive correlation between plasma level of IL-6 and age ($r=0.254$, $p<0.05$).

Table 2 Supplement. Relationship between mRNA expression of selected pro-inflammatory cytokines in blood vessels and their plasma levels.

	IMA	LAD
**TNF**	r= -0.14; p= 0.27	r= -0.10; p= 0.70
**IL-6**	r= -0.08; p= 0.51	r= 0.45; p= 0.07
**IL-1β**	r= 0.01; p= 0.92	r= 0.17; p= 0.51

The correlations between mRNA expression of TNF (TNF-α), IL-6, and IL-1β in the internal mammary arteries (IMA) and left anterior descending coronary arteries (LAD) and their concentrations in the plasma are shown. Samples were obtained from patients undergoing CABG surgery or heart transplantation (HTx). The correlations were calculated using Spearman's rank order correlation. N=69 for IMA, N=17 for LAD, CABG - Coronary artery bypass graft, HTx – Heart transplantation.

Table 3 Supplement. Relationship between mRNA expression and plasma levels of selected pro-inflammatory cytokines, vascular superoxide, and blood pressure values.

	**SBP**	**DBP**
**Vascular mRNA expression (dCT)**		
TNF	0.11	0.25*
IL-6	0.07	0.24*
IL-1β	0.14	0.24*
**Plasma protein level**		
TNF	0.14	0.19
IL-6	-0.04	0.08
IL-1β	0.06	0.08
**Vascular superoxide production**	-0.03	-0.03

Correlations between mRNA expression of TNF (TNF-α), IL-6, IL-1β in the internal mammary arteries (IMA) and their plasma levels, vascular superoxide and systolic or diastolic blood pressure (SBP, DBP, respectively). Samples were obtained from patients undergoing CABG surgery. The correlations were calculated using Spearman's rank order correlation. *P<0.05
