# Supplementary figures and images for: Systemic and local vascular inflammation and arterial reactive oxygen species generation in patients with advanced cardiovascular diseases
Source: Front Cardiovasc Med. 2023 Sep 7;10:1230051. doi: 10.3389/fcvm.2023.1230051 (PMC10513373; doi:10.3389/fcvm.2023.1230051)

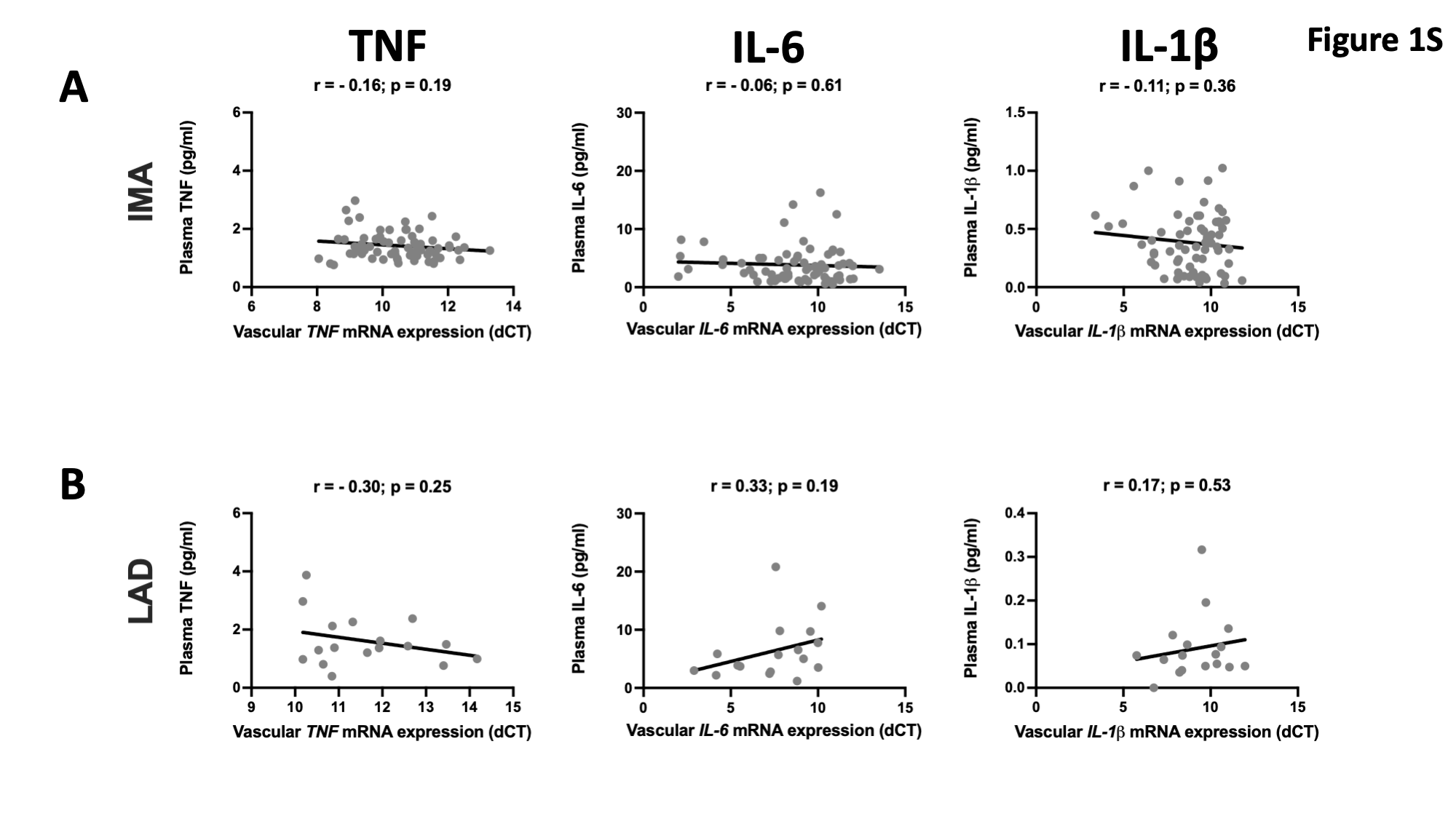

Supplement: Supplementary file 2 [file Image1.tiff]

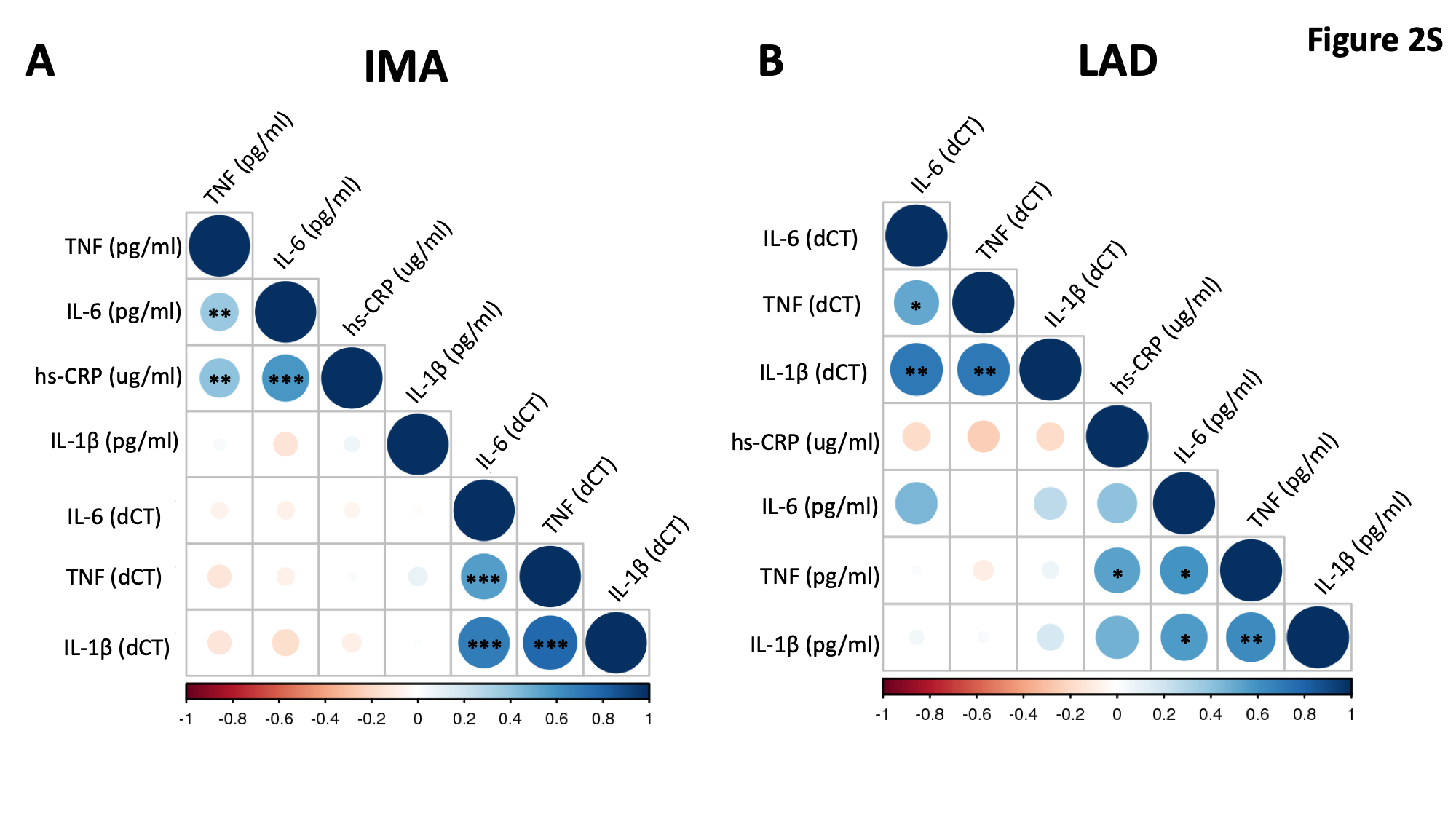

Supplement: Supplementary file 3 [file Image2.tiff]
